# Mr. Jullion, on Hydrocephalus Internus

**Published:** 1803-08-01

**Authors:** John Jullion


### To Dr. BATTY.

SIR,

MY practice in the obstetric art being deduced from the principles inculcated in your lectures on that science, I conceive it incumbent on me to transmit you the enclosed observations on a disease intimately connected with it, and in which medical practitioners in general are concerned. If the subject is favoured with insertion in your valuable Journal, from its extensive circulation I am induced to think, it will afford some gratification to a class of society whose exertions are influenced equally by the dictates of humanity as by pecuniary remuneration.

The almost invariable fatality of that alarming disease Hydrocephalus Internus, has so impressed the public mind with its attendant danger, that to determine the existence of the disease, is, in a word, to presage a short career to its possessor. Indeed, I am fearful there are some practitioners so firmly persuaded of the inefficacy of their exertions, that they resign calmly the case to Nature, who, though though in some cases capable of correcting slight morbid actions, cannot be expected to correct so great a deviation from her natural course, and more particularly as this deviation is dependent on two such important causes, increased arterial excitement, or deficient action of the absorbents. Ideas thus prejudiced, the efficacy of our art thus infringed upon, our most elaborate consideration is demanded to disperse this unmerited disgrace.

That we possess the means wherewith to accomplish this grand object will be substantiated by the case in consideration; and it is communicated with the grateful satisfaction of having restored to health, one who would otherwise have fallen a victim to this disease.

That there is a *period*, however, when these means may be had recourse to, and although vigorously pursued, yet not the desired success attend it, I think is very obvious, and the grounds of our failure may with propriety be rested on this cause. If the fluid secreted, is in that quantity which shall render an extensive introduction of mercury into the system necessary, the powers of animal life being greatly impaired, the introduction of so powerful an antidote cannot but tend to increase that debility, and in so great a degree as to hasten the termination of life before the absorption of the fluid has been affected: it is therefore highly necessary to arrest this disease in its early stage, the advantages of which are so very obvious. It would be fortunate if the exciting cause of every disease was as clearly established, the knowledge of its cause regulating our exhibition of medicines; but, if we are so determined on the nature of the disease; if the same remedies applied in two cases, equally decisive, are not attended with similar effects, to what are we to attribute this failure? Why does not the result prove equally favourable in every instance: The treatment is far from novel, it is admitted by all practitioners. These circumstances considered, this conclusion may, I think, be inferred: First, that it is a disease the *consequence* of increased arterial excitement or diminished absorption. Secondly, that our success or failure is certainly dependent on the means employed for that purpose, but particularly, on the *period* when those means are had recourse to. The first conclusion is substantiated, by the success of those means most powerfully diminishing the *volume* as well as the frequency of the blood's circulation, and those stimulants, exciting the absorption of the fluid detained by morbid action: the second, by the different success attending the application of the same remedies in cases equally decided.

With respect to the remedies employed, they will be introduced in the relation of the case; they were those generally advised. But with regard to the *period when* they are applicable, as in cases of dangerous uterine hæmorrhages, we can fix no exact limitation; as the life or death of our patient is in our hands, we must act decidedly but cautiously.

If circumstances equally favourable appear, as in this instance, on the fourth day, sufficient remission of the symptoms will have ensued as to raise our expectations, and allow a remission of our applications: This relaxation favours the restitution of the natural healthy actions; and if nutriment of a light nature is exhibited in small quantities, will enable us to exhibit the stronger tonics, as bark and vol. alkali, with advantage.

CASE.—W. Coggan, No. 23, Chandler Street, Grosvenor Square, aged 19 months, having become very emaciated, and otherwise unwell, I was requested to see him. The account I received was as follows: This child, about two months previous, had recovered from an illness which lasted seven weeks, during which time the second molaris of each side of the under jaw had protruded: No alarming symptoms appeared any farther than general emaciation: His spirits and sensibility were unimpaired. On the 4th of June, the mother informed me some *different symptoms*, from what she had before observed appeared; and in the evening a violent convulsive fit occupied him for a quarter of an hour; the child became after this gradually worse, emaciated in a great degree, and his appetite totally forsook him. It was more, however, in consequence of the *different appearances* and *deadly look* of the child, than from its sufferings, that she now applied for assistance. On the 16th of June, I found him with a quick febrile pulse, inattentive to any object approaching him, but apparently at ease when allowed to remain undisturbed: His bowels being constipated, I directed calomel gr. iij. statim exhibend. On the 18th, I found the purge had operated briskly. The sensibility however was by no means relieved; on the contrary, had in a great degree increased, being now insensible even to the most luminous object; violent screams being uttered every now and then, and even strabismus was sensible to the bye-standers, the pupil having lost all excitability to contraction.

traction. On examining the mouth, I could perceive no mark or appearance of teeth protruding through, and the circumstance of having so lately cut two of the molares, induced me to think I could afford no relief mechanically. That some mischief was arising in the brain, these symptoms sufficiently indicated; I therefore was determined to lose no time; at four P. M. I applied hirudines vj. temporibus; empl. cantharid. capiti denudato, tinct. digit. et scilla, partes æqualis, gt. vj. om. 4to. hora in quovis vehiculo, and directed unq. hyd. c. camphor. ℈j nocte utend. These were repeated the ensuing day; the irritation of the head kept up by the ung. sabinæ. On the eve of the fourth day, the effects of the hyd. were evident in the system, and the symptoms were materially alleviated: he became very restless and uneasy in any position, appeared to be under much pain with the irritation which the mercurial friction produced: the degree of insensibility almost totally disappeared, and he became irritated on the approach of any of his former playmates, even as they entered the room. The iris, though not so dilated, was not endued with much power of contraction. These favourable appearances induced me to refrain from the farther exhibition of hydrag. the leeches however were employed twice more, when the ung. sabinæ alone was continued.

On the third day of our cessation of medicine, the stomach began to resume its accustomed functions (which the introduction of mercury, by friction, admitted to be regained with greater freedom), and he became weary of bed. Tonics combined with small doses of vol. alkali, were now exhibited with daily good effects. The child has now recovered its perfect health, and within these three days cut two cuspidati of the under jaw.

In the last number of your publication, Mr. Ricards relates some appearances which took place during the convalescent state of two patients who had laboured under this disease. In the present case, as the stomach began to resume its functions, a contraction of the upper and lower extremities took place, which increased to a great degree, and induced more distress than the child had suffered during the whole disease, being solicitous for food but not capable of assisting itself. As the general health however became established, this appearance gradually disappeared. The natural disposition which all muscular fibres possess, is their tone. The flexors from their insertion, may in this instance have overcome the tone of the exteriors, and in their heir action kept the extremities in a contracted state. As the excitability of the muscular fibres became more universally increased, the two sets of muscles had their actions performed with their accustomed unison.

I am, &c.

*July* 13, 1803. JOHN JULLION.

